# Effects of Salinity and Temperature on Survival, Growth, and Food Intake of Ramshorn Snail (*Helisoma anceps*)

**DOI:** 10.1002/ece3.74343

**Published:** 2026-09-11

**Authors:** Szu‐Han Chen, Tien‐Chieh Hung

**Affiliations:** ^1^ Department of Biological and Agricultural Engineering University of California, Davis Davis California USA

**Keywords:** aquaculture application, food intake, linear mixed‐effects model, osmoregulation, salinity tolerance

## Abstract

Understanding the salinity tolerance of *Helisoma anceps* is crucial for its application in aquaculture systems, particularly at the Fish Conservation and Culture Laboratory (FCCL), where the species is used to maintain water quality in Delta and Longfin Smelt culture tanks. This study investigated the effects of salinity and temperature on the survival, growth, and food intake of *H. anceps* under non‐acclimated conditions. Adult snails were exposed to seven salinity levels (0, 2.5, 5, 7.5, 10, 12.5, and 15 ppt) at two temperatures (12°C and 16°C) for 42 days, and survival, growth, and feeding rates were analyzed using Kaplan–Meier survival analysis and linear mixed‐effects models. Results showed complete mortality at ≥ 10 ppt within 48 h, identifying a salinity tolerance limit between 7.5 and 10 ppt. Growth and food intake significantly declined with increasing salinity, while temperature modulated these effects, with higher activity and food intake observed at 16°C. Shell volume‐specific growth rate (SVSGR) was more sensitive to salinity stress than shell length‐specific growth rate (SLSGR), reflecting reduced calcification efficiency under hyperosmotic conditions. The findings classify *H. anceps* as a stenohaline species with limited osmoregulatory capacity and suggest that its optimal performance occurs within 0–5 ppt salinity at 16°C. These insights provide a physiological basis for managing *H. anceps* in smelt hatchery systems and highlight the need for careful salinity control to sustain its ecological function in aquaculture operations.

## Graphical abstract

This study examines how salinity and temperature affect the survival, growth, and food intake of the freshwater snail *Helisoma anceps* . Snails exposed to salinities of 10 ppt and above died within 48 h, while growth and food intake decreased as salinity increased. The results provide information on the environmental limits of *H. anceps* and its use under low salinity culture conditions.

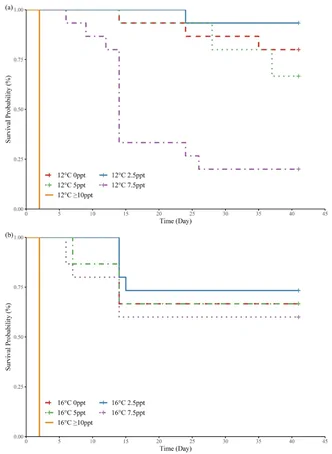

## 1 Introduction

Freshwater salinization has become an increasing environmental concern because many freshwater organisms possess limited tolerance to elevated salinity (Cañedo‐Argüelles et al. 2019; Cañedo‐Argüelles 2020; Kaushal et al. 2021). Therefore, identifying species‐specific salinity tolerance thresholds is important for understanding how freshwater organisms respond to environmental change.

Freshwater gastropods play a vital role in ecosystem stability by consuming algae, biofilms, and detritus. These feeding activities help regulate primary productivity and nutrient cycling, while also contributing to organic matter processing (Dillon 2000; Pyron and Brown 2015). As dominant members of benthic communities, they can account for a substantial portion of invertebrate biomass and significantly influence algal dynamics and nutrient flow in aquatic ecosystems (Johnson et al. 2013; Cummings et al. 2016; Mollusk Conservation Organization 2025). In practical applications, freshwater snails such as Tadpole Snail ( *Physa gyrina* ) have been shown to effectively reduce biofilm accumulation on gas exchange surfaces in Rainbow Trout ( *Oncorhynchus mykiss* ) culture systems, while also significantly lowering heterotrophic bacterial concentrations in the water column (Davidson et al. 2019).

Ramshorn snails ( *Helisoma anceps* ) are widely distributed freshwater gastropods in North America, typically inhabiting vegetation‐rich permanent water bodies such as lakes and ponds (Burch 1992; Laman et al. 1984; O'Neal and Soulliere 2006; Pip 1987). Adults generally reach a shell diameter of approximately 8–16 mm and exhibit considerable variation in shell morphology and coloration. The shell is sinistrally coiled, thick, and rounded, with distinct ridges on both the spire and umbilical sides, which give rise to the common name “two‐ridge ramshorn.” In mature individuals, the shell lip becomes thickened and flared, and shell coloration ranges from pale horn to white, often varying among populations (Burch 1989, 1992).

*Helisoma anceps* are used at the Fish Conservation and Culture Laboratory (FCCL) at the University of California, Davis, in Delta Smelt ( *Hypomesus transpacificus* ) and Longfin Smelt ( *Spirinchus thaleichthys* ) aquaculture. They are added to the larval and juvenile tanks to help maintain water quality conditions by consuming feces, algae, uneaten feed, and organic debris (Hung et al. 2018). Over 20,000 snails are employed in the tanks each year. This helps create a suitable aquatic environment for these endangered species (Hung et al. 2018; Baskerville‐Bridges et al. 2004; Lindberg et al. 2013; U.S. Fish and Wildlife Service 2023a, 2023b).

Given the critical role of *H. anceps* in both natural and controlled environments, it is essential to understand how environmental conditions, particularly salinity and temperature, influence their growth, survival, and food intake. Because *H. anceps* typically inhabit low‐salinity freshwater environments (Burch 1989), it is expected to be sensitive to elevated salinity. Previous studies have suggested that freshwater gastropods may experience osmotic challenges under elevated salinity, which could contribute to reduced growth and survival (Dillon 2000). Studies on freshwater pulmonate snails have further demonstrated that salinity can influence multiple biological responses. In *Physa acuta* , growth and reproduction showed nonlinear responses to salinity, with performance increasing at low to intermediate salinity levels and declining at higher salinities (Kefford and Nugegoda 2005). Stockwell et al. (2011) reported that increasing salinity exposure reduced survival and reproduction in *P. acuta* . More recent studies have also documented reduced movement in *Physella acuta* under elevated salinity (Wilder et al. 2024) and reduced growth in *P. acuta* under elevated salinity (Baudrix et al. 2025). Together, these studies demonstrate that salinity can influence multiple aspects of freshwater pulmonate performance, although the direction and magnitude of responses may vary among biological endpoints and salinity levels. However, the effects of salinity and temperature on *H. anceps* remain poorly understood. Therefore, further research is needed to evaluate the responses of this species under different environmental conditions and to support its effective use in conservation hatchery operations.

Temperature is another critical factor that influences the physiological state and growth performance of freshwater gastropods. By regulating metabolic processes and energy allocation, it directly affects survival, growth, and reproduction (Newell and Branch 1980; Clarke 1993). Within the optimal range, higher temperatures boost metabolic activity, enhancing growth and feeding rates. However, exceeding this range disrupts metabolic efficiency, impairs growth, increases stress, and raises mortality risk (Newell and Branch 1980). In contrast, low temperatures slow metabolic processes, reducing energy production and physiological activity. Studies on freshwater Apple Snail ( *Pomacea canaliculata* ) revealed cold environments suppress metabolic activity, leading to lower feeding rates and slower growth (Matsukura et al. 2009). Similarly, Clarke (1993) noted that low temperatures often decrease metabolic rates, energy allocation, and overall physiological functions in mollusks. These findings underscore the critical role of temperature in shaping survival, growth, and other biological responses of freshwater gastropods across diverse environmental conditions.

Currently, studies on *H. anceps* primarily focus on parasite‐related topics, whereas its responses to environmental conditions remain poorly understood. Accordingly, this study investigated the effects of salinity and temperature on the survival, growth, and food intake of *H. anceps* . We hypothesized that increasing salinity would reduce survival, growth, and food intake. We further predicted that elevated temperature would enhance food intake under tolerable salinity conditions but would not offset the detrimental effects of high salinity. By identifying the salinity tolerance threshold of *H. anceps* , this study provides information relevant to both the management of this species in conservation hatcheries and a broader understanding of freshwater gastropod responses to environmental change.

## 2 Materials and Methods

### 2.1 Snail Collection and Culture

**FIGURE 1:**
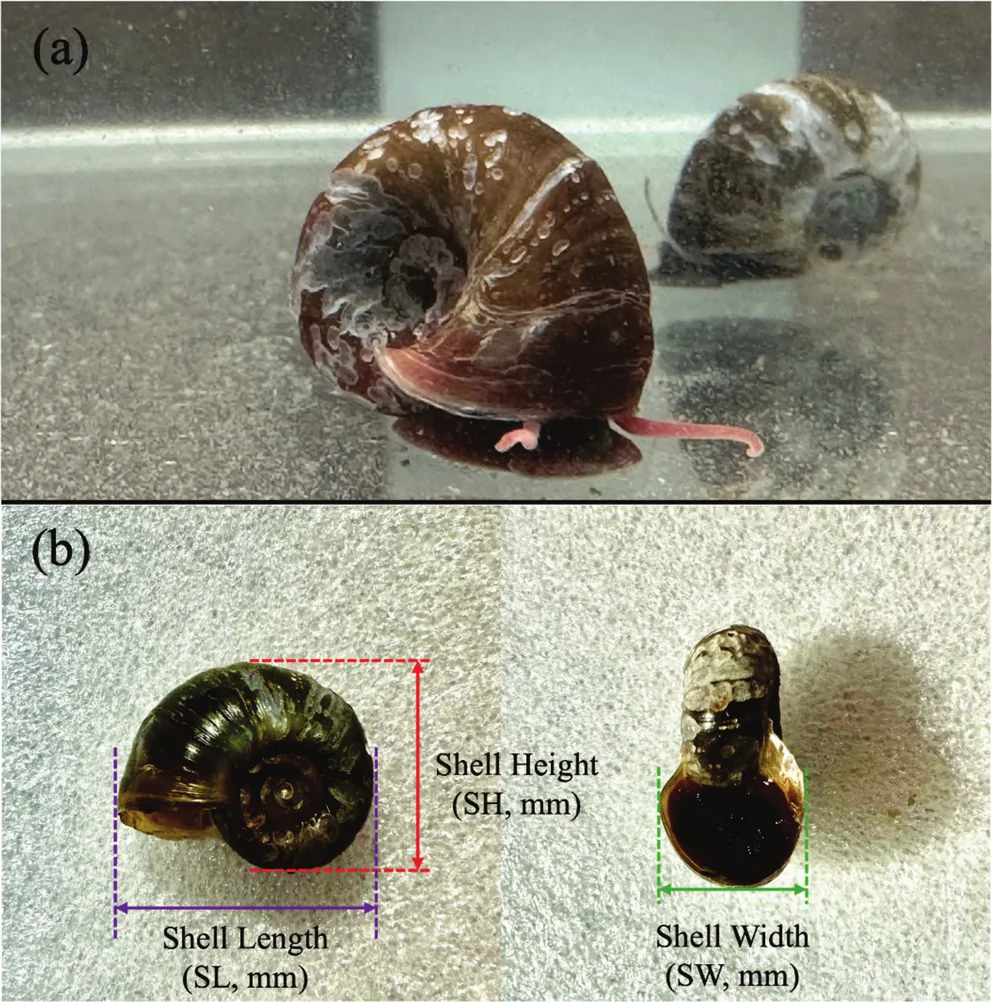
(a) Cultured ramshorn snails, *Helisoma anceps* (Menke 1830), used in this study were obtained from the captive population maintained at the UC Davis Fish Conservation and Culture Laboratory. (b) Shell measurements used in this study, including shell length (SL), shell height (SH), and shell width (SW).

A total of 240 *H. anceps* (Figure 1a) were randomly collected from a snail production tank (1.52 m in diameter and 0.61 m in depth) located in the yard of the FCCL facility and disinfected using a diluted Pond Rid‐ich solution (pre‐mixed 55 mL/gal with water, Apopka, FL, USA) to reduce the parasite load. Collected snails were immediately placed into a 20‐L bucket filled with pre‐treated channel water from the California Aqueduct. The water was treated with bead filters (Aquadyne 8000, Aquadyne, Hartwell, GA) for particle removal and UV sterilizers (ALSV‐3DX and ALQ360IL, Aqualogic, San Diego, CA, USA) for disinfection. The snails were acclimatized in the bucket for 1 week, during which they were allowed to freely lay eggs. The water in the bucket was changed daily with new water, and the bucket was maintained at 20°C in a temperature‐controlled room with a 12‐h light/12‐h dark cycle. Snails were fed with BioVita Starter Mesh feed (Bio Oregon, WA, USA) to satiation before the experiment started.

### 2.2 Experiments

#### 2.2.1 Survival

A total of 210 adult snails (0.47 ± 0.01 g in weight; 12.39 ± 0.05 mm in shell length [SL]) were selected and randomly distributed into 42,500‐mL bottles, with 5 snails per bottle. The experiment consisted of 14 salinity × temperature treatment combinations, including seven salinity levels (0, 2.5, 5, 7.5, 10, 12.5, and 15 ppt) prepared by Instant Ocean sea salt. Salinity was verified using a calibrated Pro30 conductivity/salinity meter (YSI Inc., Yellow Springs, OH, USA). Each salinity treatment was maintained at either 12°C or 16°C, with three replicate bottles per treatment combination. The experimental bottles were placed in two temperature‐controlled water tanks maintained at 12°C and 16°C, respectively, throughout the experiment. Salinity and temperature treatments were applied at the bottle level; therefore, the bottle was considered the experimental unit, while individual snails were considered observational units nested within bottles.

Survival tests followed an acute exposure design, in which individuals were transferred directly from freshwater into the target salinity without prior acclimation. This design was selected to reflect routine FCCL operating conditions, where *H. anceps* may be transferred directly from freshwater culture conditions into aquaculture systems with different salinities. The survival of the snails was monitored three times daily till the end of the study (42 days). To further confirm mortality, we followed the criteria of Liu et al. (2022). For *H. anceps* , which lacks an operculum, individuals were considered dead if they showed no movement and failed to respond to gentle stimulation of the foot region with a sharp object.

#### 2.2.2 Growth

Snails were photographed before assignment to experimental bottles and were individually identified based on their distinctive shell patterns during subsequent measurements. Body weight (BW), shell length (SL), shell height (SH), and shell width (SW) were measured (Figure 1b) and recorded weekly throughout the trial period using a Cole‐Parmer balance (TA 224.C, Cole‐Parmer, Vernon Hills, IL, USA) with a precision of 0.0001 g and a digital vernier caliper, respectively. Shell volume (SV) was estimated using a geometric approximation based on shell dimensions:

$$\text{Shell volume}\;\left(\mathrm{SV}\right)=\frac{4\pi }{3}\times \left(\frac{\mathrm{SL}}{2}\right)\times \left(\frac{\mathrm{SH}}{2}\right)\times \left(\frac{\mathrm{SW}}{2}\right)$$

The body weight specific growth rate (BWSGR) for each parameter was determined using the appropriate equations, based on the guidance from Crane et al. (2020):

$$\text{BWSGR}=100\times \left(e^{g}-1\right)$$

$$g=\frac{\ln\left(M_{2}\right)-\ln\left(M_{1}\right)}{t}$$

where *M*_1_ is the initial measurements, *M*_2_ is the final measurements, and *t* is the time interval (days).

#### 2.2.3 Food Intake

Food intake was measured at the bottle level twice a week (every Wednesday and Sunday). Because food was provided collectively to the 5 snails within each bottle and uneaten food was recovered from the bottle as a whole, individual food intake could not be determined. Therefore, each bottle provided one food‐intake observation per feeding interval and was treated as the experimental unit for food‐intake analysis.

Fish food (BioVita Starter Mesh, Bio Oregon, WA, USA) was pretreated by drying in an oven at 105°C for 48 h, following a modified procedure based on AOAC: Official Methods of Analysis to ensure consistent moisture content (Helrich 1990). The feed was weighed and provided to each bottle at the start of each feeding period (i.e., the snails were fed twice a week). Before the following feeding events, uneaten food was collected and filtered using a 600‐mL Buchner Funnel Filtering Kit (Southern Labware, Cumming, GA, USA), dried at 105°C for 24 h, and weighed. Blanks were prepared using the same process but with no snail present to correct for the dissolution of feed. The food intake rate (FIR) was calculated following the equation below:

$$\mathrm{FIR}=\frac{W_{i}-W_{f}-W_{b}}{W_{s}\times t}$$

where *W*_*i*_ is the initial food weight, *W*_*f*_ is the uneaten food weight, *W*_*b*_ is the blank value, *W*_*s*_ is the snail body weight, and *t* is the time interval (days).

### 2.3 Statistical Analyses

All statistical analyses were performed using R software (version 4.5.1, R Core Team 2025). Survival analysis was conducted using the “survival” package (Therneau 2024) and the “survminer” package (Kassambara et al. 2024). Kaplan–Meier estimation was used to compare survival probabilities among treatment groups with different temperatures and salinities. To simplify interpretation, treatment groups with salinity levels ≥ 10 ppt were combined into a single group because all snails died within 48 h and exhibited similarly rapid declines in survival. This grouping was used to simplify the presentation and interpretation of the survival patterns; the 3 salinity treatments remained distinct in the original experimental design and raw dataset. Because this grouping was determined after observing the mortality patterns, it is acknowledged as a limitation of the analysis. Survival curves were generated using the “ggsurvplot” function with customized color and linetype settings, and final figures were annotated and exported using the “cowplot” package (Wilke 2025).

For growth analysis, linear mixed‐effects models (LMMs) were fitted using the “lme4” package (Bates et al. 2015) and the “lmerTest” package (Kuznetsova et al. 2017) to evaluate the effects of salinity, temperature, and time on three key indicators: Body weight specific growth rate (BWSGR), shell length specific growth rate (SLSGR), and shell volume specific growth rate (SVSGR). Because individual snails were nested within replicate bottles and were measured repeatedly over time, bottle identity and individual identity were included as random intercepts. Bottle identity accounted for the non‐independence of individuals sharing the same experimental unit, whereas individual identity accounted for repeated measurements of the same snail over time. Random slopes for time were excluded because the model failed to converge, and a random‐intercept structure was retained for model stability. The significance of fixed effects was assessed using Type III analysis of variance (ANOVA), which provided degrees of freedom, *F*‐values, and *p*‐values for each fixed factor. Model outputs were exported for reporting and interpretation.

To further examine weekly differences among treatment groups, separate models were fitted for each week. Estimated marginal means (EMMs) for each treatment combination were calculated using the “emmeans” package (Lenth 2016). Pairwise comparisons were adjusted for multiple testing using the Sidak correction, and compact letter displays (CLDs) were generated using the “multcompView” package (Graves et al. 2024) to visualize results. The grouping letters were standardized across weeks according to the descending order of EMM values to ensure consistency and interpretability. All visualizations were created using the “ggplot2” package (Wickham 2016), with customized axis labels, color schemes, and faceting layouts to clearly illustrate the temporal trends in BWSGR, SLSGR, and SVSGR across treatment conditions.

## 3 Results

### 3.1 Survival

**FIGURE 2:**
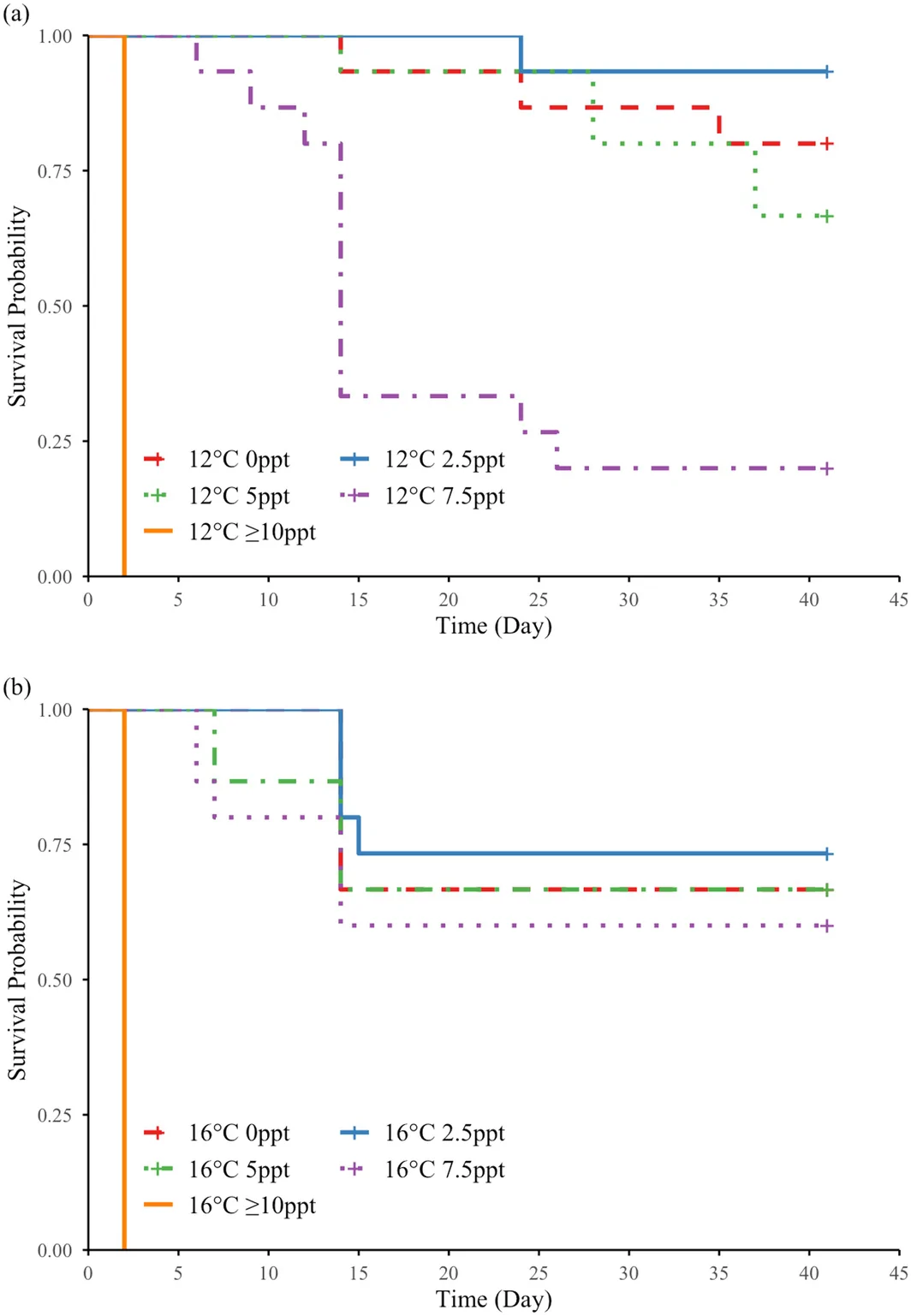
Kaplan–Meier survival curves of *H. ancep*s at (a) 12°C and (b) 16°C under varying salinity conditions. High‐salinity groups (10–15 ppt) were merged into a single category (≥ 10 ppt) in both panels to address curve overlap.

Kaplan–Meier survival curves for *Helisoma anceps* under different salinity and temperature treatments were separately plotted for 12°C (Figure 2a) and 16°C (Figure 2b). The log‐rank test revealed significant differences in survival curves among the various salinity and temperature treatments (*χ* ^2^ = 222.0, df = 13, *p* < 0.001). Under both temperature conditions, individuals in the high‐salinity groups (10, 12.5, and 15 ppt) all died within 48 h, resulting in a rapid drop in survival probability to 0%. Therefore, these three groups were combined and presented as a single “≥ 10 ppt” high‐salinity group in the figures.

Although all the high salinity groups were included in the overall Kaplan–Meier curves and global log‐rank test, statistical limitations (e.g., a singular covariance matrix) precluded them in subsequent pairwise comparisons. Pairwise analysis was thus performed only among the eight low to moderate salinity groups (0–7.5 ppt) under both temperature regimes. At 12°C, no significant difference was found among the 0, 2.5, and 5 ppt groups, and similarly, no difference was observed between the 0 and 2.5 ppt groups at 16°C. In contrast, the 7.5 ppt group at 12°C showed a significantly lower survival rate compared to the other treatments. At 16°C, although survival in the 7.5 ppt group was slightly reduced, the difference was not statistically significant.

### 3.2 Growth

#### 3.2.1 Body Weight

The BWSGR of *H. anceps* was influenced by a combination of temporal and environmental factors. Results from the linear mixed‐effects model (Table 1) indicated that time (*p* < 0.001), along with its interactions with salinity (*p* < 0.001) and temperature (*p* = 0.0414), had significant effects on the BWSGR. In contrast, temperature and salinity alone did not result in statistically significant effects.

**TABLE 1:** Results of linear mixed‐effects (LMMs) models showing the effects of temperature, salinity, week, and their interactions on body weight specific growth rate (BWSGR, %) of *H. anceps* .

Response trait	Fixed and random variables with estimates					
BWSGR (%)	*Fixed effect*	*numDF*	*denDF*	*F‐ratio*	*p*	*Effect size (η* ^*2*^ *)*
	Temperature	1	672	0.41	0.5233	0.0006
	Salinity	3	672	1.52	0.2090	0.0067
	Week	5	672	37.33	< 0.0001	0.22
	Temperature × Salinity	3	672	0.90	0.4414	0.0040
	Temperature × Week	5	672	2.33	0.0414	0.02
	Salinity × Week	15	672	4.16	< 0.0001	0.08
	Temperature × Salinity × Week	15	672	1.26	0.2255	0.03
	*Random effect*	*Variance*	SD			
	ID	0	0			
	Residuals	0.45	0.67			

*Note:* Both fixed and random effects with estimates, including effect sizes (*η* ^2^), are presented. In the model, numDF = numerator degrees of freedom; denDF = denominator degrees of freedom; SD = standard deviation. Residuals refer to the unexplained variance in the model. Significant values are italicized at the levels of *p* < 0.05, *p* < 0.01, and *p* < 0.001.

**FIGURE 3:**
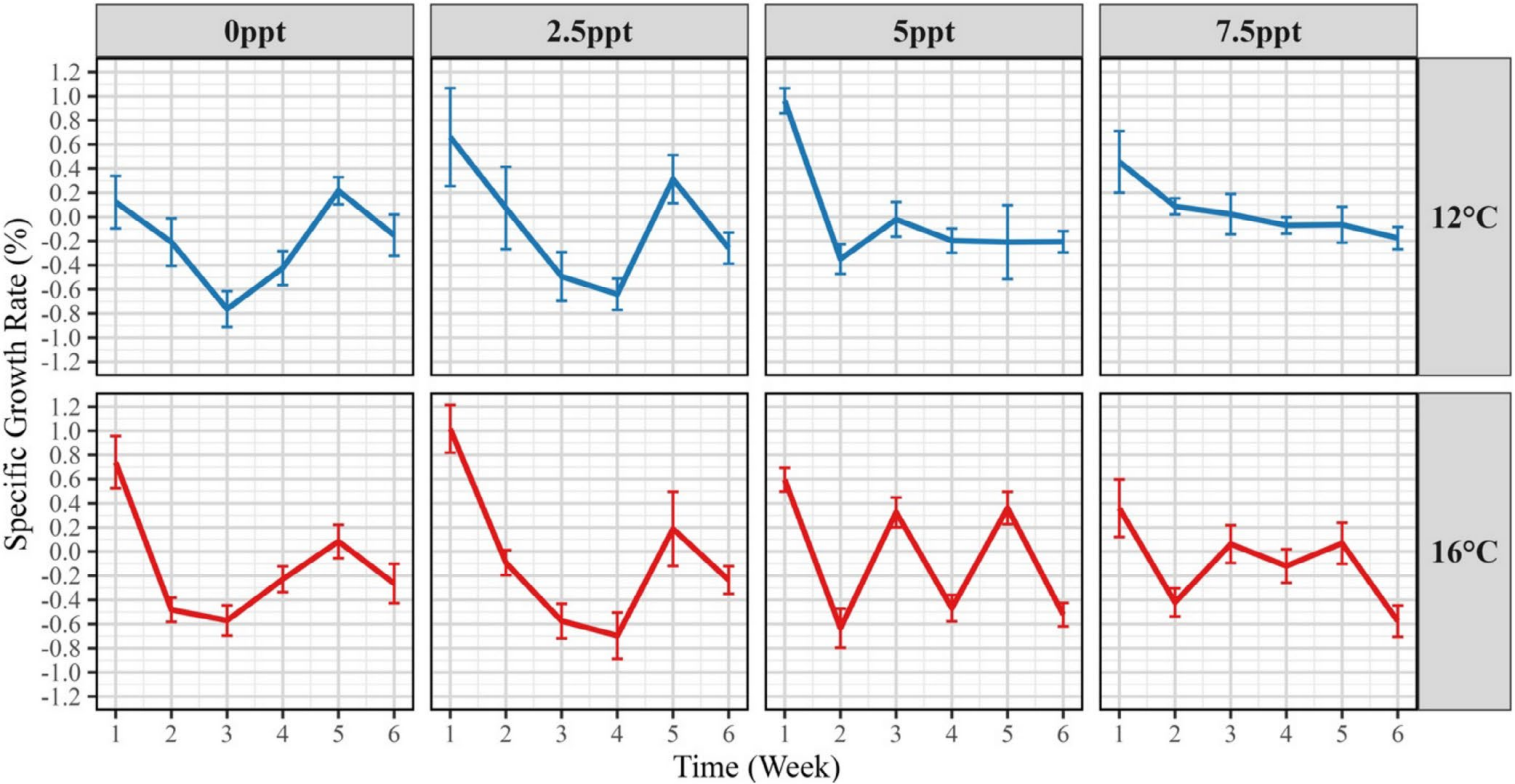
Weekly changes in body weight specific growth rate (BWSGR) of *H. ancep*s under two temperatures (12°C and 16°C) and four salinity levels (0, 2.5, 5, and 7.5 ppt). Each panel displays the mean BWSGR ± standard error over a 6‐week period. Rows represent temperature treatments; columns represent salinity levels.

As illustrated in Figure 3, BWSGR generally declined over time across all treatment groups. At 12°C, the 5 ppt group initially showed the highest BWSGR, followed by a sharp decline in subsequent weeks. The 0 and 2.5 ppt groups exhibited moderate fluctuations over time, while the 7.5 ppt group consistently maintained a relatively stable but low BWSGR throughout the 6‐week period. Similar temporal trends were observed at 16°C, although fluctuations appeared more pronounced, particularly in the 5 ppt group.

Pairwise comparisons summarized in Table S1 revealed specific intergroup differences at various time points. For example, during week 3, the 0 ppt group at 12°C had significantly lower BWSGR than the 5 ppt group (*p* < 0.05). In week 6, both the 5 and 7.5 ppt groups at 16°C exhibited significantly lower BWSGR compared to the other treatment groups. Overall, the results showed significant differences in BWSGR across time, with distinct temporal trends observed under different temperatures and salinity conditions.

#### 3.2.2 Shell Length and Volume

The SLSGR and SVSGR of *H. anceps* were affected by different combinations of environmental and temporal factors. According to the linear mixed‐effects model (Table 2), salinity had a significant effect on both SLSGR (*p* = 0.0020) and SVSGR (*p* = 0.0015). For SLSGR, significant interactions were found between temperature and time (*p* = 0.0103) and between salinity and time (*p* = 0.0048). For SVSGR, significant effects were also observed for time (*p* = 0.0138), temperature × time (*p* < 0.001), salinity × time (*p* = 0.0063), and the three‐way interaction of temperature × salinity × time (*p* = 0.0001).

**TABLE 2:** Results of linear mixed‐effects (LMMs) models showing the effects of temperature, salinity, week, and their interactions on shell length specific growth rate (SLSGR, %) and shell volume specific growth rate (SVSGR, %) of *H. anceps* .

Response trait	Fixed and random variables with estimates					
SLSGR (%)	*Fixed effect*	*numDF*	*denDF*	*F‐ratio*	*p*	*Effect size (η* ^*2*^ *)*
	Temperature	1	672	2.18	0.1406	0.0032
	Salinity	3	672	5.00	0.0020	0.02
	Week	5	672	0.92	0.4665	0.0068
	Temperature × Salinity	3	672	0.15	0.9301	0.0007
	Temperature × Week	5	672	3.03	0.0103	0.02
	Salinity × Week	15	672	2.23	0.0048	0.05
	Temperature × Salinity × Week	15	672	1.64	0.0594	0.04
	*Random effect*	*Variance*	SD			
	ID	0	0			
	Residuals	0.03	0.15			

Response trait	Fixed and random variables with estimates					
SVSGR (%)	*Fixed effect*	*numDF*	*denDF*	*F‐ratio*	*p*	*Effect size (η* ^*2*^ *)*
	Temperature	1	672	1.9222	0.1661	0.0029
	Salinity	3	672	5.1996	0.0015	0.02
	Week	5	672	2.8842	0.0138	0.02
	Temperature × Salinity	3	672	0.2953	0.8288	0.0013
	Temperature × Week	5	672	5.4337	< 0.0001	0.04
	Salinity × Week	15	672	2.1696	0.0063	0.05
	Temperature × Salinity × Week	15	672	3.0166	< 0.0001	0.06
	*Random effect*	*Variance*	SD			
	ID	0	0			
	Residuals	0.08	0.29			

*Note:* Both fixed and random effects with estimates, including effect sizes (*η* ^2^), are presented. In the model, numDF = numerator degrees of freedom; denDF = denominator degrees of freedom; SD = standard deviation. Residuals refer to the unexplained variance in the model. Significant values are italicized at the levels of *p* < 0.05, *p* < 0.01, and *p* < 0.001.

**FIGURE 4:**
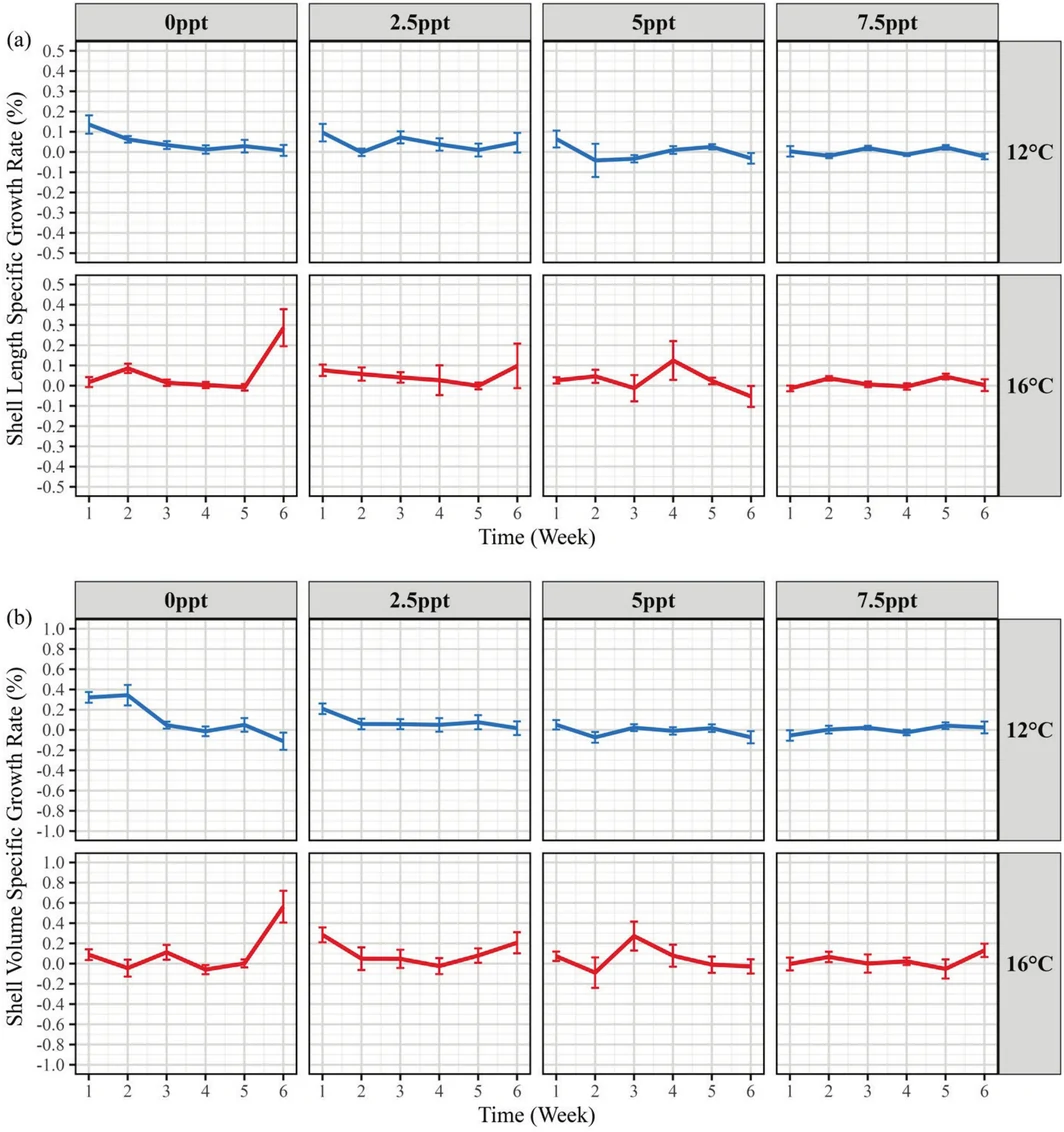
Weekly changes in (a) shell length specific growth rate (SLSGR) and (b) shell volume specific growth rate (SVSGR) of *H. anceps* under two temperatures (12°C and 16°C) and four salinity levels (0, 2.5, 5, and 7.5 ppt). Each panel displays the mean specific growth rate ± standard error over a 6‐week period. Rows represent temperature treatments; columns represent salinity levels.

As shown in Figure 4a, SLSGR exhibited limited variation across treatment groups. At 12°C, all salinity treatments showed a slight but not significant decreasing trend over time. At 16°C, the SLSGR curves were more variable, but remained within a narrow range. A moderate increase was observed in the 7.5 ppt group in week 6, but overall, temporal changes remained relatively subtle. In contrast, SVSGR values (Figure 4b) showed greater temporal and treatment‐based variation. At 12°C, the SVSGR generally declined over time across most salinity levels. At 16°C, an overall fluctuating pattern was observed, particularly in the 5 ppt group, which showed a noticeable increase in SVSGR during week 6.

The pairwise comparison results for SLSGR and SVSGR are presented in Tables S2 and S3, respectively. Although most differences among treatment groups were not statistically significant, several notable significant differences were observed. For instance, in Week 1, the SLSGR of the 12°C, 0 ppt group was significantly higher than that of the other groups, while the 16°C, 7.5 ppt group exhibited the lowest value. By Week 6, the 16°C, 0 ppt group showed the highest SLSGR, significantly exceeding the other groups. The 2.5 ppt groups at both temperatures also exhibited slightly higher values than the remaining groups and reached statistical significance, whereas the 12°C, 0 ppt group and the other remaining treatments showed poorer performance.

For SVSGR, the 12°C, 0 ppt group demonstrated the highest values during Weeks 1 and 2. However, by Week 6, the 16°C 0 ppt group outperformed all others, with the rest of the groups, which included the previously superior 12°C, 0 ppt group, showing significantly lower values. Overall, both SLSGR and SVSGR were significantly influenced by the interaction of temperature, salinity, and exposure duration, with SVSGR exhibiting more pronounced variability among treatment groups.

### 3.3 Food Intake

The FIR of *H. anceps* was significantly affected by temperature, salinity, time, and their interactions. According to the linear mixed‐effects model (Table 3), temperature (*p* < 0.001), salinity (*p* < 0.001), and time (*p* < 0.001) all had significant effects on FIR. In addition, significant interaction effects were observed for temperature × time (*p* < 0.001), salinity × time (*p* < 0.001), and temperature × salinity × time (*p* =0.3949). Corresponding effect sizes indicated a small‐to‐moderate effect of salinity (*η* ^2^ = 0.09) and temperature (*η* ^2^ = 0.09), while time and all interaction terms showed small or negligible effects (*η* ^2^ ≤ 0.03).

**TABLE 3:** Results of linear mixed‐effects (LMMs) models showing the effects of temperature, salinity, time, and their interactions on food intake rate (FIR, %) of *H. anceps* .

Response trait	Fixed and random variables with estimates					
FIR (g/g/Day)	*Fixed effect*	*numDF*	*denDF*	*F‐ratio*	*p*	*Effect size (η* ^*2*^ *)*
	Temperature	1	192	71.03	< 0.0001	0.09
	Salinity	3	192	103.00	< 0.0001	0.09
	Time	11	192	14.37	0.0819	0.0001
	Temperature × Salinity	3	192	2.27	< 0.0001	0.01
	Temperature × Time	11	192	5.46	< 0.0001	0.01
	Salinity × Time	33	192	2.75	< 0.0001	0.03
	Temperature × Salinity × Time	33	192	1.06	0.3949	0.0001
	*Random effect*	*Variance*	SD			
	ID	0.0001	0.0001			
	Residuals	0.0001	0.0001			

*Note:* Both fixed and random effects with estimates, including effect sizes (*η* ^2^), are presented. In the model, numDF = numerator degrees of freedom; denDF = denominator degrees of freedom; SD = standard deviation. Residuals refer to the unexplained variance in the model. Significant values are italicized at the levels of *p* < 0.05, *p* < 0.01, and *p* < 0.001.

**FIGURE 5:**
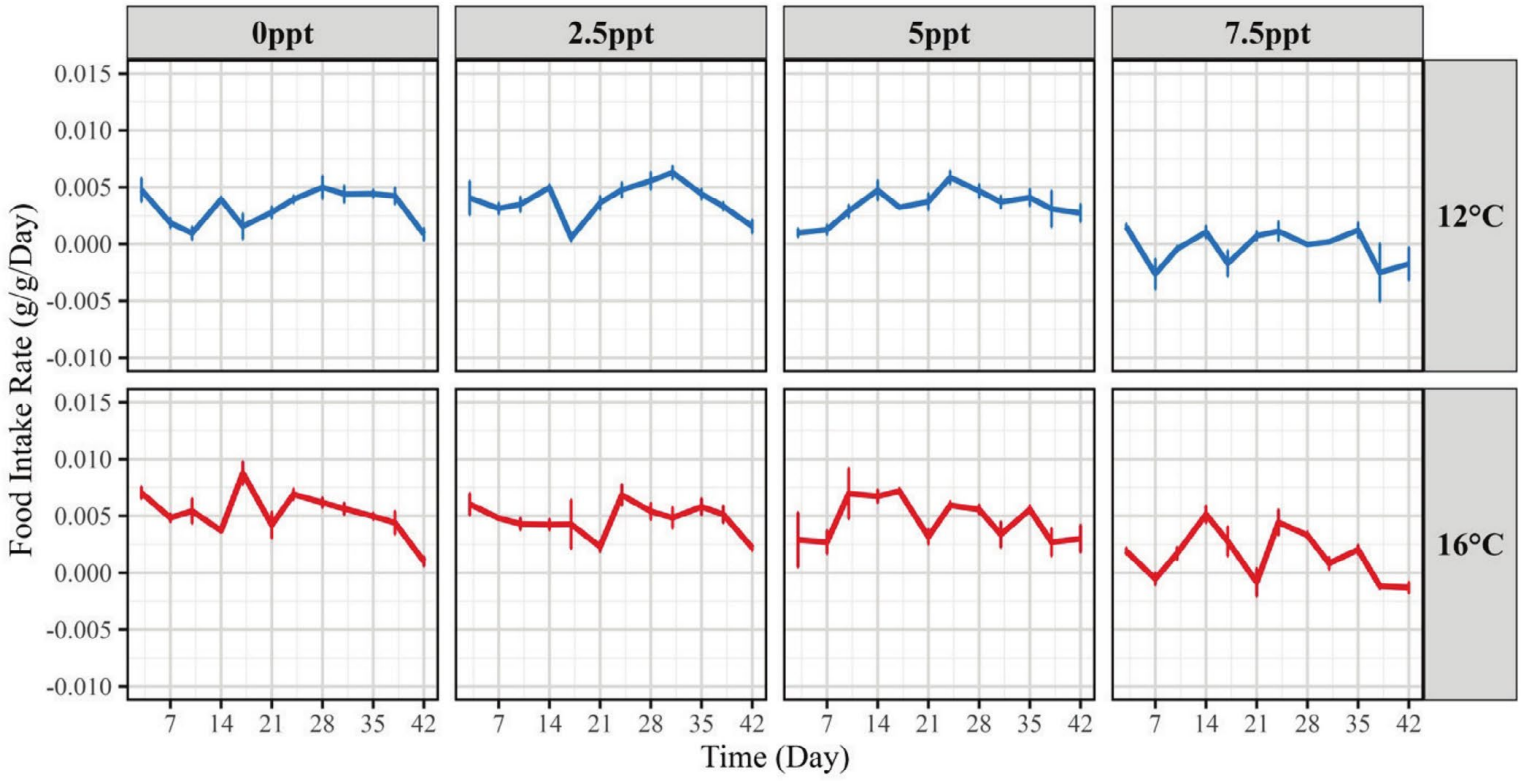
Weekly changes in food intake rate of *H. ancep*s under two temperatures (12°C and 16°C) and four salinity levels (0, 2.5, 5, and 7.5 ppt). Each panel displays the mean FIR ± standard error over a 6‐week period. Rows represent temperature treatments; columns represent salinity levels.

As shown in Figure 5, FIR varied over time across different treatments. At 12°C, the overall FIR remained low throughout the 6‐week period across all salinity levels. Among them, the 5 ppt group showed slightly elevated FIR values during the early weeks, while the 7.5 ppt group consistently exhibited the lowest values across the entire experiment.

At 16°C, FIR values were noticeably higher, particularly during the initial phase of the experiment (day 3 to day 14). The 0 and 2.5 ppt groups displayed the highest FIR peaks in the early weeks, outperforming the higher salinity groups. Over time, a general decline in FIR was observed across all treatments, especially from day 28 to day 42. The 7.5 ppt group exhibited the lowest FIR values consistently under both temperatures, especially during the latter half of the experiment.

Pairwise comparisons presented in Table S4 also revealed significant differences among treatment groups at multiple time points. Overall, the FIR of *H. anceps* was clearly influenced by the interaction between temperature and salinity. Notably, the low to moderate salinity groups (0, 2.5, and 5 ppt) at 16°C exhibited significantly higher mean FIR than their counterparts at 12°C across most sampling points, with the exception of Day 3, where no significant difference was observed. In contrast, the 7.5 ppt group at 12°C consistently showed the lowest FIR throughout the experimental period.

## 4 Discussion

Understanding the environmental thresholds of *H. anceps* is essential for optimizing its use in aquaculture systems and assessing its ecological resilience under changing water quality conditions. In the Fish Conservation and Culture Laboratory (FCCL), *H. anceps* is commonly introduced into rearing tanks of Delta smelt ( *Hypomesus transpacificus* ) and Longfin smelt ( *Spirinchus thaleichthys* ) to remove uneaten feed and maintain tank cleanliness. The two temperature regimes tested in this study (12°C and 16°C) were chosen to reflect the standard rearing temperatures used at FCCL, where Longfin smelt are typically cultured at 12°C and Delta smelt at 16°C. These temperatures represent the optimal thermal ranges for both species during captive propagation (Hung et al. 2024; Fish Conservation and Culture Laboratory 2025). Therefore, assessing the performance of *H. anceps* under these temperature conditions provides direct relevance to its practical application in smelt aquaculture operations.

This study systematically evaluated the survival, growth, and food intake of *H. anceps* under various salinity and temperature conditions. Beyond the general negative effects of elevated salinity, our results identified a transition from sublethal reductions in growth and food intake at 7.5 ppt to rapid mortality at ≥ 10 ppt. This relatively narrow transition suggests that small increases in salinity near this range may lead to substantial changes in the biological responses of *H. anceps* . In addition, temperature‐dependent differences in food intake suggest that responses to salinity may vary with thermal conditions. These findings provide species‐specific information relevant to understanding how freshwater gastropods may respond to increasing freshwater salinization and interacting environmental changes. In terms of survival, all *H. anceps* individuals exposed to salinities ≥ 10 ppt died within 2 days, regardless of temperature. Log‐rank analysis confirmed significant differences in survival rates among treatment groups, with the estimated upper salinity tolerance threshold falling between 7.5 and 10 ppt. Although the 10, 12.5, and 15 ppt treatments could have been retained separately, they were grouped post hoc because all individuals died within 48 h and exhibited similarly rapid mortality patterns. Therefore, the ≥ 10 ppt category represents a common acute high‐salinity response rather than identical effects among the three salinity levels. This result aligns with findings from studies on the freshwater Apple Snail, which exhibited high mortality at salinities of 6–8 ppt (Qin et al. 2020). Liu et al. (2022) further reported that *P. canaliculata* individuals, at all developmental stages, perished within 9 days when exposed to ≥ 7.5 PSU (practical salinity unit) salinity, suggesting a salinity tolerance limit around 5 PSU. Similarly, the closely related species Spiketop Applesnail ( *Pomacea bridgesi* ) exhibits constrained osmoregulatory responses at elevated salinity, consistent with osmoconforming behavior (Jordan and Deaton 1999). Studies on freshwater pulmonate snails provide additional context for these responses. Stockwell et al. (2011) reported that increasing salinity reduced survival and reproduction in *P. acuta* , while Kefford and Nugegoda (2005) found nonlinear responses of growth and reproduction to salinity, with performance increasing at low to intermediate salinity levels and declining at higher salinities. More recent studies have also reported reduced movement in *P. acuta* under elevated salinity (Wilder et al. 2024) and reduced growth in *P. acuta* under elevated salinity (Baudrix et al. 2025). Together, these studies indicate that salinity can affect multiple biological responses in freshwater pulmonate snails, although the magnitude and pattern of these effects vary among species, endpoints, and salinity conditions.

The South American freshwater snail *Chilina dombeiana* can only survive in environments with ≤ 8 PSU salinity. Despite its capacity for aerial respiration, this species exhibited neither escape behaviors nor acclimatory responses under high‐salinity conditions, indicating limitations in both physiological and behavioral adaptation (Barrios‐Figueroa and Urbina 2023). These observations are consistent with our findings, where *H. anceps* exhibited rapid mortality under acute high‐salinity exposure, suggesting limited tolerance to abrupt salinity stress. Similar patterns have been reported for other freshwater invertebrates; for instance, Berezina (2003) documented mass mortality in various taxa at salinities of 5–8 ppt. These studies support the notion that freshwater snails are highly sensitive to abrupt salinity changes under non‐acclimated conditions. The current study adopted a direct‐exposure design, in which individuals were transferred directly from freshwater into the target salinity environment without prior acclimation. This approach simulates the common operating procedures of the FCCL, where snails are typically introduced into aquaculture systems with various salinities without acclimation. Thus, the observed physiological responses should be interpreted as context‐specific limitations under “non‐acclimated” conditions, rather than as absolute tolerance thresholds. Nonetheless, the direct exposure method holds practical value for simulating real‐world aquaculture operations and assessing potential risks.

In our study, BWSGR exhibited a clear time‐dependent decline across all temperature‐salinity combinations. One possible explanation for this pattern is an energetic trade‐off under prolonged environmental stress. Barrios‐Figueroa and Urbina (2023) observed reductions in oxygen consumption, ammonia excretion, and muscle water content in *C. dombeiana* exposed to elevated salinity, suggesting physiological adjustments under salinity stress. As proposed by Calow (1985), environmental stress may increase energetic demands for maintenance and thereby reduce the energy available for growth. Thus the sustained reduction in SGR observed in the present study is consistent with a possible shift in energy allocation toward maintenance; however, energy allocation and metabolic processes were not directly measured.

In particular, the 5 ppt group showed relatively higher SGR in the early phase of the experiment, especially at 12°C, but this advantage diminished later. In contrast, the 7.5 ppt group exhibited consistently low SGR throughout the trial period, indicating that even moderately elevated salinity may exert long‐term suppressive effects on growth. Similar patterns were reported in Apple Snail, where survival and growth declined at 6–8 ppt salinity (Qin et al. 2020), reinforcing the notion that salinity stress negatively affects somatic growth in freshwater gastropods.

This study demonstrates that the SVSGR of *H. anceps* is more responsive to environmental changes than the SLSGR. While both growth indices were significantly affected by salinity, SVSGR exhibited more pronounced temporal fluctuations, particularly under 16°C, where differences among treatment groups became more evident. In contrast, SLSGR showed relatively minor variation among groups and remained more stable over time, whereas shell volume, as a three‐dimensional growth parameter, appeared to be more sensitive to changes in physiological stress.

The shell of mollusks primarily grows through the process of calcification, in which mantle epithelial cells secrete calcium carbonate and organic matrix to build the shell structure (Hiebenthal et al. 2012; Jahromi and Barzkar 2018). However, salinity stress may inhibit this calcification process, thereby reducing shell formation efficiency in gastropods and other calcifying organisms (Bashevkin and Pechenik 2015). This mechanism may help explain why SVSGR is particularly sensitive to high salinity treatments (e.g., 7.5 ppt). This interpretation is consistent with the findings of Goodfriend (1986), who noted that shell morphology exhibits high environmental plasticity, and with Palmer (1992), who emphasized that shell formation involves considerable metabolic costs, especially in increasing volume and thickness. As such, SVSGR may serve as a more sensitive indicator of growth responses to environmental stress than shell length alone.

In addition, this study found that high salinity, particularly at 7.5 ppt, significantly suppressed SVSGR of *H. anceps* , aligning with findings by Qin et al. (2020) who reported that elevated salinity imposes physiological stress and inhibits growth in freshwater snails. Notably, although the 12°C and 0 ppt group initially exhibited the highest growth performance, its advantage diminished over time and was ultimately surpassed by the 16°C and 0 ppt group. This suggests that *H. anceps* may maintain more stable growth at 16°C under low salinity conditions during prolonged exposure.

The FIR was also significantly influenced by temperature and salinity. At most time points during the experiment, FIR values at 12°C were markedly lower than those at 16°C across all salinity levels, indicating that *H. anceps* exhibits better food intake at 16°C than 12°C across salinity levels. This observation is supported by previous studies; for instance, Zhang et al. (2018) demonstrated that higher temperatures increased herbivory and animal prey consumption in the omnivorous Great Pond Snail *Lymnaea stagnalis* . Similarly, Miyata and Nakatsubo (2024) found that the feeding rate of Apple Snail increased with rising temperature, which they associated with increased metabolic activity and feeding demand.

However, as the experiment progressed, FIR showed a general decline across all treatment groups, with the most pronounced decrease observed in the 7.5 ppt group. Liu et al. (2022) reported that Apple Snail exhibited almost no feeding activity under high salinity conditions (7.5 and 10 ppt), which is consistent with the present study, where snails at salinities beyond their suitable range (≥ 7.5 ppt) often remained retracted within their shells and showed minimal activity. This reduced activity and prolonged shell retraction may reflect a behavioral response to unfavorable salinity conditions, although physiological impairment under osmotic stress, reduced motivation to forage, or a general deterioration in condition could also have contributed to the observed reduction in food intake. These findings align with those of Qin et al. (2020), who suggested that osmotic imbalance can impair feeding behavior and digestive function in freshwater gastropods.

In summary, *H. anceps* exhibits limited salinity tolerance, with significant reductions in growth and feeding observed at 7.5 ppt, and complete mortality occurring at salinities of 10 ppt or higher. These findings support its potential as a functional species for removing uneaten feed in aquaculture systems, but only under low to moderate salinity conditions. While *H. anceps* maintained relatively high survival, growth, and food intake within the 0–5 ppt range, its long‐term physiological responses and regulatory mechanisms remain to be further investigated. This study also revealed an interaction between temperature and salinity, highlighting the need for careful environmental management in semi‐intensive aquaculture systems, as elevated salinity not only reduces survival but may also compromise its ecological role in waste removal. It is therefore recommended that salinity be maintained between 0 and 5 ppt for optimal performance. Future studies should explore the potential for long‐term or transgenerational acclimation and assess the effects of other environmental factors (such as pH) on its physiology and behavior. If salinity tolerance can be enhanced through adaptation or selective breeding, its applicability in recirculating or flow‐through systems could be greatly expanded, and effective environmental management will be essential to ensuring a stable ecological role for *H. anceps* in integrated aquaculture systems.

## Author Contributions

**Szu‐Han Chen:** conceptualization (supporting), data curation (lead), formal analysis (lead), methodology (equal), writing – original draft (lead), writing – review and editing (equal). **Tien‐Chieh Hung:** conceptualization (lead), funding acquisition (lead), methodology (equal), project administration (lead), supervision (lead), writing – review and editing (equal).

## Funding

This work was supported by the Bureau of Reclamation (10.13039/100006450), R20AC00027, Department of Water Resources (10.13039/100004813), 4600015779.

## Conflicts of Interest

The authors declare no conflicts of interest.

## Supporting information


**Data S1:** Supporting Information.

**Table S1:** Estimated marginal means (EMMs), standard errors (SE), degrees of freedom (df), 95% confidence intervals (CI), and significance groupings (Sig.) for the body weight specific growth rate (BWSGR) of *Helisoma anceps* under different temperature (12°C and 16°C) and salinity (0–7.5 ppt) treatments across six experimental weeks. Results were derived from linear mixed‐effects models (LMMs) and post hoc pairwise comparisons adjusted by the Sidak correction.
**Table S2:** Estimated marginal means (EMMs), standard errors (SE), degrees of freedom (df), 95% confidence intervals (CI), and significance groupings (Sig.) for the shell length specific growth rate (SLSGR) of *Helisoma anceps* under different temperature (12°C and 16°C) and salinity (0–7.5 ppt) treatments across six experimental weeks. Results were obtained using linear mixed‐effects models (LMMs) followed by pairwise comparisons adjusted with the Sidak method.
**Table S3:** Estimated marginal means (EMMs), standard errors (SE), degrees of freedom (df), 95% confidence intervals (CI), and significance groupings (Sig.) for the shell volume specific growth rate (SVSGR) of *Helisoma anceps* under different temperature (12°C and 16°C) and salinity (0–7.5 ppt) treatments across six experimental weeks. Statistical analyses were performed using linear mixed‐effects models (LMMs) and the Sidak‐adjusted pairwise comparisons.
**Table S4:** Estimated marginal means (EMMs), standard errors (SE), degrees of freedom (df), 95% confidence intervals (CI), and significance groupings (Sig.) for the food intake rate (FIR) of *Helisoma anceps* under different temperature (12°C and 16°C) and salinity (0–7.5 ppt) treatments across six experimental weeks. Results were obtained from linear mixed‐effects models (LMMs) followed by pairwise comparisons corrected with the Sidak adjustment.

## Data Availability

All the required data are uploaded as Supporting Information.
