# Supplementary material for: Effects of Salinity and Temperature on Survival, Growth, and Food Intake of Ramshorn Snail (Helisoma anceps)
Source: Ecol Evol. 2026 Sep 11;16(9):e74343. doi: 10.1002/ece3.74343 (PMC13564242; doi:10.1002/ece3.74343)
Supplement: Supplementary file 1 [file ECE3-16-e74343-s002.docx]

Table S1. Estimated marginal means (EMMs), standard errors (S.E.), degrees of freedom (df), 95% confidence intervals (CI), and significance groupings (Sig.) for the body weight specific growth rate (BWSGR) of *Helisoma anceps* under different temperature (12°C and 16°C) and salinity (0–7.5 ppt) treatments across six experimental weeks. Results were derived from linear mixed-effects models (LMMs) and post hoc pairwise comparisons adjusted by the Sidak correction.

| Week | Temperature  (°C) | Salinity  (ppt) | EMMs | S.E. | df | 95% CI | Sig. |
| --- | --- | --- | --- | --- | --- | --- | --- |
| 1 | 12 | 0 | 0.1219 | 0.2345 | 112 | (-0.530, 0.774) | a |
|  |  | 2.5 | 0.6611 | 0.2345 |  | (0.009, 1.313) | a |
|  |  | 5 | 0.9614 | 0.2345 |  | (0.310, 1.613) | a |
|  |  | 7.5 | 0.4560 | 0.2345 |  | (-0.196, 1.108) | a |
|  | 16 | 0 | 0.7400 | 0.2345 | 112 | (0.088, 1.392) | a |
|  |  | 2.5 | 1.0174 | 0.2345 |  | (0.366, 1.669) | a |
|  |  | 5 | 0.5950 | 0.2345 |  | (-0.057, 1.247) | a |
|  |  | 7.5 | 0.3590 | 0.2345 |  | (-0.293, 1.011) | a |
| 2 | 12 | 0 | -0.2089 | 0.1713 | 112 | (-0.685, 0.267) | a |
|  |  | 2.5 | 0.0735 | 0.1713 |  | (-0.403, 0.550) | a |
|  |  | 5 | -0.3496 | 0.1713 |  | (-0.826, 0.127) | a |
|  |  | 7.5 | 0.0876 | 0.1713 |  | (-0.389, 0.564) | a |
|  | 16 | 0 | -0.4823 | 0.1713 | 112 | (-0.958, -0.006) | a |
|  |  | 2.5 | -0.0921 | 0.1713 |  | (-0.568, 0.384) | a |
|  |  | 5 | -0.6345 | 0.1713 |  | (-1.111, -0.158) | a |
|  |  | 7.5 | -0.4208 | 0.1713 |  | (-0.897, 0.055) | a |
| 3 | 12 | 0 | -0.7633 | 0.1527 | 112 | (-1.188, -0.339) | d |
|  |  | 2.5 | -0.4938 | 0.1527 |  | (-0.918, -0.069) | c |
|  |  | 5 | -0.0207 | 0.1527 |  | (-0.445, 0.404) | b |
|  |  | 7.5 | 0.0232 | 0.1527 |  | (-0.401, 0.448) | b |
|  | 16 | 0 | -0.5707 | 0.1527 | 112 | (-0.995, -0.146) | c |
|  |  | 2.5 | -0.5747 | 0.1527 |  | (-0.999, -0.150) | c |
|  |  | 5 | 0.3251 | 0.1527 |  | (-0.099, 0.750) | a |
|  |  | 7.5 | 0.0619 | 0.1527 |  | (-0.362, 0.486) | b |
| 4 | 12 | 0 | -0.4238 | 0.1277 | 112 | (-0.779, -0.069) | b |
|  |  | 2.5 | -0.6408 | 0.1277 |  | (-0.996, -0.286) | b |
|  |  | 5 | -0.1968 | 0.1277 |  | (-0.552, 0.158) | b |
|  |  | 7.5 | -0.0701 | 0.1277 |  | (-0.425, 0.285) | a |
|  | 16 | 0 | -0.2293 | 0.1277 | 112 | (-0.584, 0.126) | b |
|  |  | 2.5 | -0.6966 | 0.1277 |  | (-1.052, -0.342) | c |
|  |  | 5 | -0.4690 | 0.1277 |  | (-0.824, -0.114) | b |
|  |  | 7.5 | -0.1198 | 0.1277 |  | (-0.475, 0.235) | a |
| 5 | 12 | 0 | 0.2163 | 0.2028 | 112 | (-0.347, 0.780) | a |
|  |  | 2.5 | 0.3124 | 0.2028 |  | (-0.251, 0.876) | a |
|  |  | 5 | -0.2091 | 0.2028 |  | (-0.773, 0.355) | a |
|  |  | 7.5 | -0.0648 | 0.2028 |  | (-0.629, 0.499) | a |
|  | 16 | 0 | 0.0834 | 0.2028 | 112 | (-0.480, 0.647) | a |
|  |  | 2.5 | 0.1875 | 0.2028 |  | (-0.376, 0.751) | a |
|  |  | 5 | 0.3612 | 0.2028 |  | (-0.203, 0.925) | a |
|  |  | 7.5 | 0.0681 | 0.2028 |  | (-0.496, 0.632) | a |
| 6 | 12 | 0 | -0.1516 | 0.1265 | 112 | (-0.503, 0.200) | a |
|  |  | 2.5 | -0.2591 | 0.1265 |  | (-0.611, 0.093) | a |
|  |  | 5 | -0.2062 | 0.1265 |  | (-0.558, 0.145) | a |
|  |  | 7.5 | -0.1755 | 0.1265 |  | (-0.527, 0.176) | a |
|  | 16 | 0 | -0.2643 | 0.1265 | 112 | (-0.616, 0.087) | a |
|  |  | 2.5 | -0.2357 | 0.1265 |  | (-0.587, 0.116) | a |
|  |  | 5 | -0.5224 | 0.1265 |  | (-0.874, -0.171) | a |
|  |  | 7.5 | -0.5759 | 0.1265 |  | (-0.928, -0.224) | a |

Table S2. Estimated marginal means (EMMs), standard errors (S.E.), degrees of freedom (df), 95% confidence intervals (CI), and significance groupings (Sig.) for the shell length specific growth rate (SLSGR) of *Helisoma anceps* under different temperature (12°C and 16°C) and salinity (0–7.5 ppt) treatments across six experimental weeks. Results were obtained using linear mixed-effects models (LMMs) followed by pairwise comparisons adjusted with the Sidak method.

| Week | Temperature  (°C) | Salinity  (ppt) | EMMs | S.E. | df | 95% CI | Sig. |
| --- | --- | --- | --- | --- | --- | --- | --- |
| 1 | 12 | 0 | 0.1357 | 0.0319 | 112 | (0.047, 0.224) | a |
|  |  | 2.5 | 0.0956 | 0.0319 |  | (0.007, 0.184) | b |
|  |  | 5 | 0.0640 | 0.0319 |  | (-0.025, 0.153) | b |
|  |  | 7.5 | 0.0032 | 0.0319 |  | (-0.086, 0.092) | b |
|  | 16 | 0 | 0.0177 | 0.0319 | 112 | (-0.071, 0.106) | b |
|  |  | 2.5 | 0.0762 | 0.0319 |  | (-0.013, 0.165) | b |
|  |  | 5 | 0.0261 | 0.0319 |  | (-0.063, 0.115) | b |
|  |  | 7.5 | -0.0137 | 0.0319 |  | (-0.102, 0.075) | c |
| 2 | 12 | 0 | 0.0624 | 0.0357 | 112 | (-0.037, 0.162) | a |
|  |  | 2.5 | -0.0017 | 0.0357 |  | (-0.101, 0.098) | a |
|  |  | 5 | -0.0417 | 0.0357 |  | (-0.141, 0.058) | a |
|  |  | 7.5 | -0.0193 | 0.0357 |  | (-0.119, 0.080) | a |
|  | 16 | 0 | 0.0854 | 0.0357 | 112 | (-0.014, 0.185) | a |
|  |  | 2.5 | 0.0575 | 0.0357 |  | (-0.042, 0.157) | a |
|  |  | 5 | 0.0462 | 0.0357 |  | (-0.053, 0.145) | a |
|  |  | 7.5 | 0.0361 | 0.0357 |  | (-0.063, 0.135) | a |
| 3 | 12 | 0 | 0.0341 | 0.0296 | 112 | (-0.048, 0.116) | a |
|  |  | 2.5 | 0.0723 | 0.0296 |  | (-0.010, 0.155) | a |
|  |  | 5 | -0.0335 | 0.0296 |  | (-0.116, 0.049) | a |
|  |  | 7.5 | 0.0195 | 0.0296 |  | (-0.063, 0.102) | a |
|  | 16 | 0 | 0.0147 | 0.0296 | 112 | (-0.068, 0.097) | a |
|  |  | 2.5 | 0.0409 | 0.0296 |  | (-0.041, 0.123) | a |
|  |  | 5 | -0.0124 | 0.0296 |  | (-0.095, 0.070) | a |
|  |  | 7.5 | 0.0064 | 0.0296 |  | (-0.076, 0.089) | a |
| 4 | 12 | 0 | 0.0121 | 0.0459 | 112 | (-0.115, 0.140) | a |
|  |  | 2.5 | 0.0371 | 0.0459 |  | (-0.090, 0.165) | a |
|  |  | 5 | 0.0098 | 0.0459 |  | (-0.118, 0.137) | a |
|  |  | 7.5 | -0.0129 | 0.0459 |  | (-0.140, 0.115) | a |
|  | 16 | 0 | 0.0030 | 0.0459 | 112 | (-0.124, 0.131) | a |
|  |  | 2.5 | 0.0269 | 0.0459 |  | (-0.101, 0.154) | a |
|  |  | 5 | 0.1245 | 0.0459 |  | (-0.003, 0.252) | a |
|  |  | 7.5 | -0.0045 | 0.0459 |  | (-0.132, 0.123) | a |
| 5 | 12 | 0 | 0.0288 | 0.0202 | 112 | (-0.028, 0.085) | a |
|  |  | 2.5 | 0.0097 | 0.0202 |  | (-0.047, 0.066) | a |
|  |  | 5 | 0.0255 | 0.0202 |  | (-0.031, 0.082) | a |
|  |  | 7.5 | 0.0228 | 0.0202 |  | (-0.033, 0.079) | a |
|  | 16 | 0 | -0.0078 | 0.0202 | 112 | (-0.064, 0.049) | a |
|  |  | 2.5 | -0.0011 | 0.0202 |  | (-0.057, 0.055) | a |
|  |  | 5 | 0.0233 | 0.0202 |  | (-0.033, 0.080) | a |
|  |  | 7.5 | 0.0449 | 0.0202 |  | (-0.011, 0.101) | a |
| 6 | 12 | 0 | 0.0078 | 0.0592 | 112 | (-0.157, 0.172) | c |
|  |  | 2.5 | 0.0461 | 0.0592 |  | (-0.118, 0.211) | b |
|  |  | 5 | -0.0314 | 0.0592 |  | (-0.196, 0.133) | c |
|  |  | 7.5 | -0.0224 | 0.0592 |  | (-0.187, 0.142) | c |
|  | 16 | 0 | 0.2865 | 0.0592 | 112 | (0.122, 0.451) | a |
|  |  | 2.5 | 0.0979 | 0.0592 |  | (-0.067, 0.262) | b |
|  |  | 5 | -0.0533 | 0.0592 |  | (-0.218, 0.111) | c |
|  |  | 7.5 | 0.0024 | 0.0592 |  | (-0.162, 0.167) | c |

Table S3. Estimated marginal means (EMMs), standard errors (S.E.), degrees of freedom (df), 95% confidence intervals (CI), and significance groupings (Sig.) for the shell volume specific growth rate (SVSGR) of *Helisoma anceps* under different temperature (12°C and 16°C) and salinity (0–7.5 ppt) treatments across six experimental weeks. Statistical analyses were performed using linear mixed-effects models (LMMs) and the Sidak-adjusted pairwise comparisons.

| Week | Temperature  (°C) | Salinity  (ppt) | EMMs | S.E. | df | 95% CI | Sig. |
| --- | --- | --- | --- | --- | --- | --- | --- |
| 1 | 12 | 0 | 0.3217 | 0.0556 | 112 | (0.167, 0.476) | a |
|  |  | 2.5 | 0.2092 | 0.0556 |  | (0.055, 0.364) | c |
|  |  | 5 | 0.0512 | 0.0556 |  | (-0.103, 0.206) | e |
|  |  | 7.5 | -0.0549 | 0.0556 |  | (-0.209, 0.100) | g |
|  | 16 | 0 | 0.0882 | 0.0556 | 112 | (-0.066, 0.243) | d |
|  |  | 2.5 | 0.2846 | 0.0556 |  | (0.130, 0.439) | b |
|  |  | 5 | 0.0729 | 0.0556 |  | (-0.082, 0.227) | d |
|  |  | 7.5 | -0.0037 | 0.0556 |  | (-0.158, 0.151) | f |
| 2 | 12 | 0 | 0.3435 | 0.0881 | 112 | (0.099, 0.588) | a |
|  |  | 2.5 | 0.0585 | 0.0881 |  | (-0.186, 0.303) | b |
|  |  | 5 | -0.0734 | 0.0881 |  | (-0.318, 0.171) | c |
|  |  | 7.5 | 0.0030 | 0.0881 |  | (-0.242, 0.248) | b |
|  | 16 | 0 | -0.0447 | 0.0881 | 112 | (-0.289, 0.200) | b |
|  |  | 2.5 | 0.0487 | 0.0881 |  | (-0.196, 0.294) | b |
|  |  | 5 | -0.0893 | 0.0881 |  | (-0.334, 0.156) | c |
|  |  | 7.5 | 0.0662 | 0.0881 |  | (-0.179, 0.311) | b |
| 3 | 12 | 0 | 0.0476 | 0.0767 | 112 | (-0.166, 0.261) | a |
|  |  | 2.5 | 0.0570 | 0.0767 |  | (-0.156, 0.270) | a |
|  |  | 5 | 0.0224 | 0.0767 |  | (-0.191, 0.236) | a |
|  |  | 7.5 | 0.0222 | 0.0767 |  | (-0.191, 0.235) | a |
|  | 16 | 0 | 0.1117 | 0.0767 | 112 | (-0.102, 0.325) | a |
|  |  | 2.5 | 0.0470 | 0.0767 |  | (-0.166, 0.260) | a |
|  |  | 5 | 0.2724 | 0.0767 |  | (0.059, 0.486) | a |
|  |  | 7.5 | 0.0011 | 0.0767 |  | (-0.212, 0.214) | a |
| 4 | 12 | 0 | -0.0137 | 0.0614 | 112 | (-0.184, 0.157) | a |
|  |  | 2.5 | 0.0500 | 0.0614 |  | (-0.121, 0.221) | a |
|  |  | 5 | -0.0098 | 0.0614 |  | (-0.180, 0.161) | a |
|  |  | 7.5 | -0.0250 | 0.0614 |  | (-0.196, 0.146) | a |
|  | 16 | 0 | -0.0592 | 0.0614 | 112 | (-0.230, 0.111) | a |
|  |  | 2.5 | -0.0245 | 0.0614 |  | (-0.195, 0.146) | a |
|  |  | 5 | 0.0784 | 0.0614 |  | (-0.092, 0.249) | a |
|  |  | 7.5 | 0.0224 | 0.0614 |  | (-0.148, 0.193) | a |
| 5 | 12 | 0 | 0.0500 | 0.0650 | 112 | (-0.131, 0.231) | a |
|  |  | 2.5 | 0.0764 | 0.0650 |  | (-0.104, 0.257) | a |
|  |  | 5 | 0.0176 | 0.0650 |  | (-0.163, 0.198) | a |
|  |  | 7.5 | 0.0421 | 0.0650 |  | (-0.139, 0.223) | a |
|  | 16 | 0 | 0.0019 | 0.0650 | 112 | (-0.179, 0.183) | a |
|  |  | 2.5 | 0.0793 | 0.0650 |  | (-0.101, 0.260) | a |
|  |  | 5 | -0.0105 | 0.0650 |  | (-0.191, 0.170) | a |
|  |  | 7.5 | -0.0527 | 0.0650 |  | (-0.233, 0.128) | a |
| 6 | 12 | 0 | -0.1118 | 0.0891 | 112 | (-0.359, 0.136) | c |
|  |  | 2.5 | 0.0173 | 0.0891 |  | (-0.230, 0.265) | c |
|  |  | 5 | -0.0724 | 0.0891 |  | (-0.320, 0.175) | c |
|  |  | 7.5 | 0.0247 | 0.0891 |  | (-0.223, 0.272) | c |
|  | 16 | 0 | 0.5629 | 0.0891 | 112 | (0.315, 0.811) | a |
|  |  | 2.5 | 0.2065 | 0.0891 |  | (-0.041, 0.454) | b |
|  |  | 5 | -0.0279 | 0.0891 |  | (-0.276, 0.220) | c |
|  |  | 7.5 | 0.1306 | 0.0891 |  | (-0.117, 0.378) | c |

Table S4. Estimated marginal means (EMMs), standard errors (S.E.), degrees of freedom (df), 95% confidence intervals (CI), and significance groupings (Sig.) for the food intake rate (FIR) of *Helisoma anceps* under different temperature (12°C and 16°C) and salinity (0 - 7.5 ppt) treatments across six experimental weeks. Results were obtained from linear mixed-effects models (LMMs) followed by pairwise comparisons corrected with the Sidak adjustment.

| Time  (Day) | Temperature  (°C) | Salinity  (ppt) | EMMs | S.E. | df | 95% CI | Sig. |
| --- | --- | --- | --- | --- | --- | --- | --- |
| 3 | 12 | 0 | 0.0048 | 0.0011 | 16 | (0.001, 0.008) | a |
|  |  | 2.5 | 0.0041 | 0.0011 |  | (0.000, 0.008) | a |
|  |  | 5 | 0.0010 | 0.0011 |  | (-0.003, 0.005) | a |
|  |  | 7.5 | 0.0015 | 0.0011 |  | (-0.002, 0.005) | a |
|  | 16 | 0 | 0.0070 | 0.0011 | 16 | (0.003, 0.011) | a |
|  |  | 2.5 | 0.0060 | 0.0011 |  | (0.002, 0.010) | a |
|  |  | 5 | 0.0029 | 0.0011 |  | (-0.001, 0.007) | a |
|  |  | 7.5 | 0.0019 | 0.0011 |  | (-0.002, 0.006) | a |
| 7 | 12 | 0 | 0.0019 | 0.0007 | 16 | (0.000, 0.004) | b |
|  |  | 2.5 | 0.0031 | 0.0007 |  | (0.001, 0.005) | b |
|  |  | 5 | 0.0013 | 0.0007 |  | (-0.001, 0.003) | b |
|  |  | 7.5 | -0.0026 | 0.0007 |  | (-0.005, 0.000) | d |
|  | 16 | 0 | 0.0048 | 0.0007 | 16 | (0.003, 0.007) | a |
|  |  | 2.5 | 0.0048 | 0.0007 |  | (0.003, 0.007) | a |
|  |  | 5 | 0.0027 | 0.0007 |  | (0.001, 0.005) | b |
|  |  | 7.5 | -0.0005 | 0.0007 |  | (-0.003, 0.002) | c |
| 10 | 12 | 0 | 0.0010 | 0.0010 | 16 | (-0.002, 0.004) | d |
|  |  | 2.5 | 0.0035 | 0.0010 |  | (0.000, 0.007) | c |
|  |  | 5 | 0.0029 | 0.0010 |  | (0.000, 0.006) | c |
|  |  | 7.5 | -0.0004 | 0.0010 |  | (-0.004, 0.003) | e |
|  | 16 | 0 | 0.0054 | 0.0010 | 16 | (0.002, 0.009) | b |
|  |  | 2.5 | 0.0043 | 0.0010 |  | (0.001, 0.007) | c |
|  |  | 5 | 0.0070 | 0.0010 |  | (0.004, 0.010) | a |
|  |  | 7.5 | 0.0017 | 0.0010 |  | (-0.001, 0.005) | c |
| 14 | 12 | 0 | 0.0039 | 0.0005 | 16 | (0.002, 0.006) | c |
|  |  | 2.5 | 0.0050 | 0.0005 |  | (0.003, 0.007) | b |
|  |  | 5 | 0.0047 | 0.0005 |  | (0.003, 0.006) | b |
|  |  | 7.5 | 0.0011 | 0.0005 |  | (-0.001, 0.003) | e |
|  | 16 | 0 | 0.0037 | 0.0005 | 16 | (0.002, 0.005) | d |
|  |  | 2.5 | 0.0042 | 0.0005 |  | (0.003, 0.006) | b |
|  |  | 5 | 0.0067 | 0.0005 |  | (0.005, 0.008) | a |
|  |  | 7.5 | 0.0051 | 0.0005 |  | (0.003, 0.007) | b |
| 17 | 12 | 0 | 0.0016 | 0.0011 | 16 | (-0.002, 0.005) | d |
|  |  | 2.5 | 0.0006 | 0.0011 |  | (-0.003, 0.004) | e |
|  |  | 5 | 0.0032 | 0.0011 |  | (0.000, 0.007) | c |
|  |  | 7.5 | -0.0017 | 0.0011 |  | (-0.005, 0.002) | e |
|  | 16 | 0 | 0.0088 | 0.0011 | 16 | (0.005, 0.012) | a |
|  |  | 2.5 | 0.0043 | 0.0011 |  | (0.001, 0.008) | c |
|  |  | 5 | 0.0072 | 0.0011 |  | (0.004, 0.011) | b |
|  |  | 7.5 | 0.0028 | 0.0011 |  | (-0.001, 0.006) | c |
| 21 | 12 | 0 | 0.0028 | 0.0008 | 16 | (0.000, 0.005) | b |
|  |  | 2.5 | 0.0036 | 0.0008 |  | (0.001, 0.006) | a |
|  |  | 5 | 0.0037 | 0.0008 |  | (0.001, 0.006) | a |
|  |  | 7.5 | 0.0007 | 0.0008 |  | (-0.002, 0.003) | b |
|  | 16 | 0 | 0.0042 | 0.0008 | 16 | (0.002, 0.007) | a |
|  |  | 2.5 | 0.0023 | 0.0008 |  | (0.000, 0.005) | b |
|  |  | 5 | 0.0032 | 0.0008 |  | (0.001, 0.006) | b |
|  |  | 7.5 | -0.0008 | 0.0008 |  | (-0.003, 0.002) | c |

| Time  (Day) | Temperature  (°C) | Salinity  (ppt) | EMMs | S.E. | df | 95% CI | Sig. |
| --- | --- | --- | --- | --- | --- | --- | --- |
| 24 | 12 | 0 | 0.0039 | 0.0007 | 16 | (0.002, 0.006) | b |
|  |  | 2.5 | 0.0048 | 0.0007 |  | (0.002, 0.007) | a |
|  |  | 5 | 0.0059 | 0.0007 |  | (0.004, 0.008) | a |
|  |  | 7.5 | 0.0011 | 0.0007 |  | (-0.001, 0.003) | c |
|  | 16 | 0 | 0.0069 | 0.0007 | 16 | (0.005, 0.009) | a |
|  |  | 2.5 | 0.0068 | 0.0007 |  | (0.004, 0.009) | a |
|  |  | 5 | 0.0059 | 0.0007 |  | (0.004, 0.008) | a |
|  |  | 7.5 | 0.0044 | 0.0007 |  | (0.002, 0.007) | b |
| 28 | 12 | 0 | 0.0050 | 0.0006 | 16 | (0.003, 0.007) | a |
|  |  | 2.5 | 0.0056 | 0.0006 |  | (0.004, 0.008) | a |
|  |  | 5 | 0.0047 | 0.0006 |  | (0.003, 0.007) | a |
|  |  | 7.5 | 0.0000 | 0.0006 |  | (-0.002, 0.002) | b |
|  | 16 | 0 | 0.0062 | 0.0006 | 16 | (0.004, 0.008) | a |
|  |  | 2.5 | 0.0054 | 0.0006 |  | (0.003, 0.007) | a |
|  |  | 5 | 0.0056 | 0.0006 |  | (0.004, 0.008) | a |
|  |  | 7.5 | 0.0033 | 0.0006 |  | (0.001, 0.005) | a |
| 31 | 12 | 0 | 0.0044 | 0.0007 | 16 | (0.002, 0.007) | a |
|  |  | 2.5 | 0.0063 | 0.0007 |  | (0.004, 0.009) | a |
|  |  | 5 | 0.0037 | 0.0007 |  | (0.001, 0.006) | b |
|  |  | 7.5 | 0.0002 | 0.0007 |  | (-0.002, 0.002) | e |
|  | 16 | 0 | 0.0056 | 0.0007 | 16 | (0.003, 0.008) | a |
|  |  | 2.5 | 0.0048 | 0.0007 |  | (0.003, 0.007) | a |
|  |  | 5 | 0.0034 | 0.0007 |  | (0.001, 0.006) | c |
|  |  | 7.5 | 0.0008 | 0.0007 |  | (-0.001, 0.003) | d |
| 35 | 12 | 0 | 0.0044 | 0.0005 | 16 | (0.003, 0.006) | b |
|  |  | 2.5 | 0.0044 | 0.0005 |  | (0.003, 0.006) | b |
|  |  | 5 | 0.0041 | 0.0005 |  | (0.002, 0.006) | b |
|  |  | 7.5 | 0.0012 | 0.0005 |  | (0.000, 0.003) | d |
|  | 16 | 0 | 0.0050 | 0.0005 | 16 | (0.003, 0.007) | a |
|  |  | 2.5 | 0.0058 | 0.0005 |  | (0.004, 0.007) | a |
|  |  | 5 | 0.0056 | 0.0005 |  | (0.004, 0.007) | a |
|  |  | 7.5 | 0.0020 | 0.0005 |  | (0.000, 0.004) | c |
| 38 | 12 | 0 | 0.0042 | 0.0013 | 16 | (0.000, 0.008) | b |
|  |  | 2.5 | 0.0033 | 0.0013 |  | (-0.001, 0.007) | b |
|  |  | 5 | 0.0031 | 0.0013 |  | (-0.001, 0.007) | b |
|  |  | 7.5 | -0.0025 | 0.0013 |  | (-0.006, 0.001) | c |
|  | 16 | 0 | 0.0044 | 0.0013 | 16 | (0.000, 0.008) | a |
|  |  | 2.5 | 0.0051 | 0.0013 |  | (0.001, 0.009) | a |
|  |  | 5 | 0.0027 | 0.0013 |  | (-0.001, 0.007) | b |
|  |  | 7.5 | -0.0012 | 0.0013 |  | (-0.005, 0.003) | b |
| 42 | 12 | 0 | 0.0009 | 0.0008 | 16 | (-0.002, 0.003) | b |
|  |  | 2.5 | 0.0016 | 0.0008 |  | (-0.001, 0.004) | b |
|  |  | 5 | 0.0027 | 0.0008 |  | (0.000, 0.005) | a |
|  |  | 7.5 | -0.0017 | 0.0008 |  | (-0.004, 0.001) | c |
|  | 16 | 0 | 0.0010 | 0.0008 | 16 | (-0.002, 0.004) | b |
|  |  | 2.5 | 0.0022 | 0.0008 |  | (0.000, 0.005) | b |
|  |  | 5 | 0.0030 | 0.0008 |  | (0.000, 0.006) | a |
|  |  | 7.5 | -0.0013 | 0.0008 |  | (-0.004, 0.001) | b |
